# A novel arabinose-inducible promoter in the carotenogenic yeast Xanthophyllomyces dendrorhous

**DOI:** 10.1007/s00253-026-13957-0

**Published:** 2026-07-23

**Authors:** Oscar Ulises García-Flores, Rodolfo Marsch, Alejandra Sánchez-Licona, Teresa Ponce-Noyola, Luis Bernardo Flores-Cotera

**Affiliations:** Department of Biotechnology and Bioengineering, Cinvestav-IPN, Av. Instituto Politécnico Nacional 2508, Col. San Pedro Zacatenco, Mexico City, 07360 México

**Keywords:** Inducible promoters, *EYFP* expression, Astaxanthin, Carotenoids

## Abstract

**Abstract:**

*Xanthophyllomyces dendrorhous* is a basidiomycetous yeast capable of synthesizing astaxanthin, a powerful antioxidant carotenoid pigment. Despite appreciable research efforts, molecular tools for the genetic manipulation of this yeast remain limited. Inducible promoters are essential tools to control gene expression in response to external stimuli, such as molecules or stress signals. This regulation enables precise control of gene expression in synthetic biological systems, minimizing unwanted effects caused by constant gene expression. In the present work inducible promoters from the L-arabinitol dehydrogenase (*LAD*), isocitrate lyase (*ICL*), alpha glucosidase (*AG*), glycerol kinase (*GUT1*), and xylitol dehydrogenase (*XDH*) genes were screened and evaluated. The promoter sequences were cloned into a plasmid containing the encoding sequence for Enhanced Yellow Fluorescent Protein (*EYFP*) for functional assessment. Promoter activity was quantified by fluorescence under induction and repression conditions in the engineered yeast cells. Among the promoters analyzed, only the *LAD* promoter led to high fluorescence under induction conditions, and low under repression. Yeast cells harboring the *LAD* promoter showed mostly an increasing fluorescence with culture time. Moreover, dose-response analyses with arabinose from 0.05 to 30 g/L indicated that only 2.5 g/L arabinose is needed to achieve maximum fluorescence from the yeast cells. Finally, we performed a two-stage culture of *X. dendrorhous* harboring the *LAD* promoter and arabinose as inducer for prospective novel practical applications. Overall, we believe that the *LAD* promoter is a promising tool for fine-tuning of protein expression in *X. dendrorhous*.

**Key points:**

- *A native arabinose-inducible promoter is disclosed in the yeast.*
- *LAD promoter exhibits a sensitive response to the inducer.*
- *A two-stage culture of X. dendrorhous allows first the accumulation biomass, and then the expression of EYFP.*

**Supplementary Information:**

The online version contains supplementary material available at https://doi.org/10.1007/s00253-026-13957-0.

## Introduction

*Xanthophyllomyces dendrorhous* (asexual state *Phaffia rhodozyma*) is a basidiomycetous yeast known for its capacity to synthesize astaxanthin, a potent antioxidant carotenoid pigment (Rodriguez et al. 2010). Astaxanthin protects *X. dendrorhous* cells against reactive oxygen species (ROS) such as superoxide ion (O_2_^_^), singlet oxygen (^1^O_2_), and hydrogen peroxide (H_2_O_2_) (Schroeder and Johnson 1995; Luna-Flores et al. 2023). Owing to its remarkable antioxidant properties, astaxanthin is widely utilized in aquaculture, food, pharmaceutical, and cosmetic industries (Miao et al. 2011; Davinelli et al. 2018). The yeast is capable of metabolizing a wide range of carbon sources (Palágyi et al. 2001), which exhibits a highly active acetyl-CoA metabolism and accumulates a high lipid content. These metabolic features, combined with its natural ability to produce astaxanthin, position *X. dendrorhous* as an attractive microbial platform for carotenoid biosynthesis and biotechnological exploitation.

Numerous studies have been conducted in the past to increase the cellular content of astaxanthin in *X. dendrorhous*; however, the levels attained are still low. Native strains typically accumulate 200–300 µg astaxanthin/g dry cell weight (DCW), whereas mutant strains have been reported to reach levels as high as 8500 µg/g DCW (Misawa 2021). Even so, developing new strategies to get astaxanthin-overproducing strains remains crucial at least for economic reasons. Three main approaches have been employed to enhance cellular astaxanthin accumulation: (i) optimization of culture conditions including use of fed-batch processes and alternative substrates, developing chemically defined mediums, or through exposure of yeast cells to light stress or toxic substances, or working at different pH and agitation; (ii) development and screening of overproducing mutants by mutagenic treatments with ultraviolet light, nitrosoguanidine, or methyl methane sulfonate; and (iii) genetic engineering aimed mostly on the overexpression or knockout of key genes to improve availability of substrates for astaxanthin synthesis (Hara et al. 2014b).

Genetic engineering requires tools such as episomal and integrative plasmids, a wide set of promoters, editing techniques, and transformation methods to develop novel strains with improved characteristics. Some of these tools are already available for *X. dendrorhous*, but RNA pol III specifically, and inducible promoters have barely been studied. Hara et al. 2014b, a evaluated 13 constitutive promoters and found that the alcohol dehydrogenase promoter was the strongest compared to the other reported promoters. Subsequently, these constitutive promoters have been successfully used by several researchers, e.g., for expressing genes involved in carotenoid synthesis. More recently, Tobin et al. 2024 used a transcriptomic approach to identify novel constitutive and light-inducible promoters in *X. dendrorhous*. They exposed *X. dendrorhous* cells to four different light wavelengths, and through a transcriptomic analysis, 26 promoters were identified comprising: 10 constitutive, 10 inducible, and six repressible, thus expanding the repertoire of regulatory sequences available for genetic engineering in this yeast. Nevertheless, inducible promoters responsive to other stimuli remain unexplored in this yeast. Moreover, in other yeast species, a range of promoters activated by environmental or metabolic signals have been reported, such as those induced by cold‑shock, distinctive carbon sources, lack or accumulation of essential amino acids, and ion concentration (Weinhandl et al. 2014; Johns et al. 2016; Bartolo-Aguilar et al. 2022). For certain applications, carbon-source-induced promoters are particularly advantageous because they enable maximum biomass accumulation before recombinant protein expression is induced (Weinhandl et al. 2014). For example, the *AOX* promoter is induced by ethanol in *Pichia pastoris* (Cregg et al. 1989), while the *GAL1* and *GAL2* promoters are induced with galactose in *Saccharomyces cerevisiae* (Rajeshkannan et al. 2022), or the *ICL* promoter of the basidiomycetous yeast *Rhodotorula toruloides*, which is induced by ethanol (Johns et al. 2016).

Inducible promoters have a wide range of applications in genetic engineering. They have been used to control the expression of recombinant proteins in *S. cerevisiae* and *P. pastoris*, particularly to express proteins that are toxic to cells (Weinhandl et al. 2014). In contrast, the constitutive expression of metabolic pathways can lead to decreased cell growth. This negative result may arise from two primary causes: the toxicity of the product or the unwanted redirection of cellular resources crucial for cell growth, e.g., carbon flow, redox cofactors, or ATP (Peng et al. 2015). Therefore, it may often be more desirable to activate a given metabolic pathway, using a regulated promoter, just after growth has culminated.

*X. dendrorhous* is a nonconventional yeast used for commercial astaxanthin production but with significant potential for other novel biotechnological applications. The yeast metabolizes a variety of carbon sources, so that it contains within its genome a set of well-regulated carbon-inducible promoters. Therefore, in this study, we identified and evaluated five carbon-inducible promoters using *EYFP* as reporter. Among the promoters evaluated, only the *LAD* promoter showed markedly high fluorescence under induction conditions. Finally, we developed a two-stage culture using yeast cells carrying the *LAD* promoter, to enable a process comprising two distinct phases (i) for biomass accumulation and (ii) for protein expression. We believe this promoter represents a valuable tool for forthcoming genetic engineering and biotechnological applications.

## Material and methods

### Strains, media, nucleic acids, and reagents

*Escherichia coli* TOP 10 cells were used as host for DNA preservation and propagation. The *E. coli* strains containing the constructions were grown in LB medium (g/L): tryptone 10, yeast extract 5, sodium chloride 5, and supplemented with kanamycin or ampicillin (each at 100 µg/mL).

In this work, the CBS 6938 strain of *X. dendrorhous* was used and cultivated in different media according to the intended objective. The yeast was preserved in YM-agar medium containing (g/L): glucose 10, peptone 5, yeast extract 3, malt extract 3, and agar 20. Moreover, the *X. dendrorhous* transformants carrying the studied promoters were seeded on YPD-agar containing (g/L): glucose 20, yeast extract 10, peptone 20, agar 20, and supplemented with zeocin (150 µg/mL). Also, the engineered strains containing the promoters were grown on YM medium, where glucose was replaced by either arabinose, ethanol, glycerol, acetate, maltose, xylose, or xylitol, depending on the specific engineered strain.

Plasmids used: pAG413GPD-ccdB-EYFP (Addgene #14334), pXDrDNA (developed in this work), and pJET 1.2 blunt (Thermo Scientific, Waltham, MA, USA). Enzymes and kits: Phusion High Fidelity DNA Polymerase (Thermo Scientific, Waltham, MA, USA), Clean and Concentrator Kit (Zymo Research, Irvine, CA, USA), DNeasy Plant Mini Kit (Qiagen, Hilden, Germany), and restriction enzymes and DNA ligase (Thermo Scientific, Waltham, MA, USA). Primers (Sigma-Aldrich, St. Louis, MO, USA). Antibiotics (Thermo Scientific, Waltham, MA, USA), culture medium (J.T. Baker, Center Valley, PA, USA), and substrates (Thermo Scientific, St. Louis, MO, USA).

### Molecular methods and plasmid construction

Genomic DNA was isolated from *X. dendrorhous* cells using the DNeasy Plant Mini kit according to the manufacturers’ protocol with the following modifications: cell lysis was aided by the addition of 100 µL of 0.5 mm glass beads (Thermo Scientific, Waltham, MA, USA), and the elution water was preheated to 65 °C. All DNA methods, including extraction and related techniques, were performed according to Sambrook and Russell (2001).

**Fig. 1:**
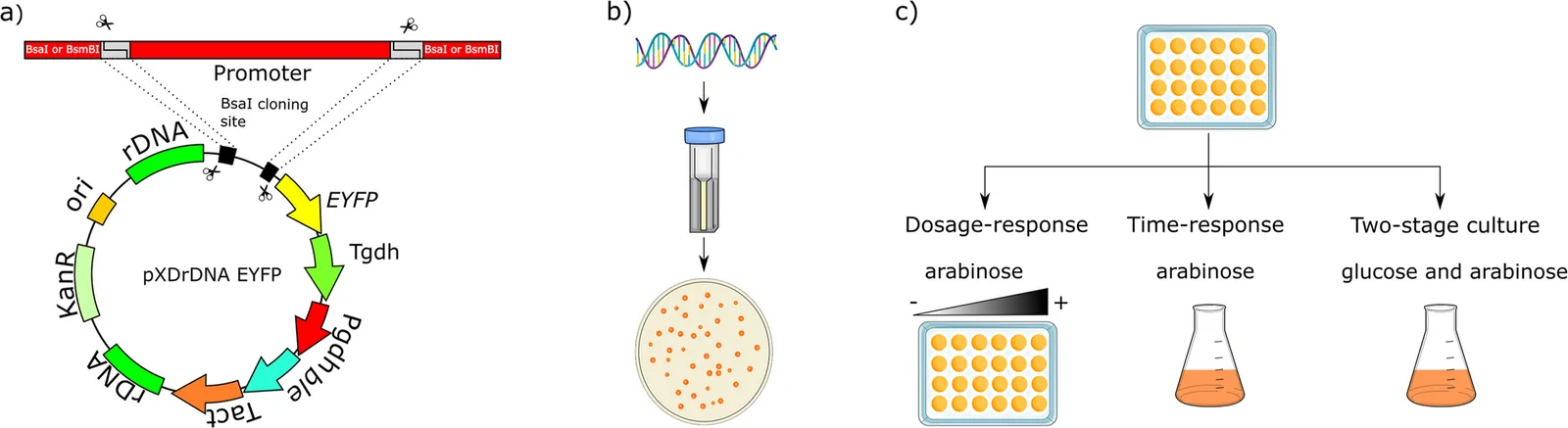
Type IIS cloning strategy for identifying and characterizing inducible promoters of *X. dendrorhous*. **a** The pXDrDNA EYFP plasmid contains a BsaI cloning site, rDNA homology arms, ble cassette resistance, ori, and KanR. The promoter was amplified with primers to add either BsaI or BsmBI sites, next, the BsaI site was then used for cloning. **b** Transformation of *X. dendrorhous*. The construction harboring the putative promoter sequence was amplified with rDNA F/R primer pair, the PCR product was purified, and the cells were transformed by electroporation. The cells were recovered and incubated for 5 h and finally plated on medium supplemented with zeocin. **c** Evaluation and characterization of the LAD promoter. The first evaluation was carried out in microplate culture, followed by an assessment of dose–response and time-dependence. Finally, a two-stage culture was performed

Primers, promoter sequences, and the annotated pXDrDNA are listed in Supplementary Tables S1, S2, and S3, respectively. The plasmid pXDrDNA-EYFP (Fig. 1a) was assembled to evaluate the screened promoters. It contains a modular sequence based on Type IIS cloning (Engler et al. 2008) and *EYFP* as reporter. The plasmid was built as follows: first, the *EYFP* gene was amplified from pAG413GPD-ccdB-EYFP using the EYFP F/R primer pair. Next, the *GDH* terminator (*GDH*-T) was amplified from *X. dendrorhous* genomic DNA using the GDH-T F/R primer pair. These fragments were then purified and fused by overlapping extension PCR, and the resulting fused fragment was cloned into the pJET 1.2 Blunt plasmid to generate pJET EYFP-GDH-T. Finally, the EYFP-GDH-T insert was subcloned from pJET-EYFP to pXDrDNA using NotI and XbaI restriction sites.

The *LAD*, *XDH*, and *ICL* promoters were amplified, purified, and cloned into the pXDrDNA-EYFP plasmid in a reaction mixture consisting of BsaI (0.5 µL), T4 DNA ligase (0.5 µL), 10X ligase buffer (2 µL), pXDrDNA-EYFP (100 ng), promoter at 5:1 insert/plasmid molar ratio, and nuclease-free water up to 20 µL. The Golden Gate Assembly–type reaction was performed in a thermal cycler as follows: 5 min at 37 °C, 5 min at 22 °C, and 5 min repeated 10 times, followed by inactivation for 10 min at 60 °C.

To clone promoter sequences (*AG* and *GUT1*) containing internal *BsaI* restriction sites, a modified Type IIS cloning strategy was employed. Instead of *BsaI*, the Type IIS *BsmBI* site was incorporated into the promoter fragments. This approach generated the identical sticky ends compatible with the pXDrDNA-EYFP cloning site. The pXDrDNA-EYFP vector and the *BsmBI*-flanked promoter fragments were digested in separate reactions, followed by enzyme inactivation and ligation. After the assembly, a total reaction mixture volume of 5 µL was used to transform *E. coli* cells.

### X. dendrorhous transformation

The transformation of *X. dendrorhous* cells was performed using DNA obtained by PCR amplification with the F/R rDNA primer pair. These primers flank the rDNA sequences, excluding ori and *KanR*, thus preventing the integration of additional sequences. Finally, the PCR-derived amplicons were purified using the Clean and Concentrator Kit.

Transformation was performed via electroporation of spheroplasts prepared according to Stowers et al. (1995). Spheroplasts were mixed with 0.5 μg of DNA in a 0.2-cm electroporation cuvette and subjected to electroporation using a Micro Pulser electroporator (Bio-Rad, Hercules, CA, USA, model 165–2100) at 2 kV.

Following electroporation, cells were recovered in 1 mL of YPD medium and incubated at 22 °C for 5 h. Cells were subsequently plated on YPD medium supplemented with zeocin to select transformants (Fig. 1b). Colonies were observed after 3–5 days of incubation. To confirm successful transformation, five colonies were randomly selected from each promoter construct, and the integration of the *EYFP* gene was verified by PCR amplification (supplementary material Figure S1).

### Promoter induction and fluorescence measurement

The expression level of *EYFP* driven by the examined promoters was initially evaluated in micro-cultures using the engineered *X. dendrorhous* strains (each carrying one promoter) and the native strain of *X. dendrorhous* (control). The native and engineered strains were grown (48 h) under repression conditions. Following, 0.1 mL of the whole yeast broth was placed into 24-well plates containing either induction or repression medium (2 mL). Following, a 3-mm glass-bead was added into each well to prevent biomass settling and to improve oxygen transfer. All plates were incubated at 22 °C and 150 rpm for 48 h. Before measuring fluorescence, each sample was washed twice with sterile water to remove residual medium and resuspended, and then fluorescence and absorbance were read. Fluorescence measurements were performed using a microplate reader (BMG LABTECH, Ortenberg, Germany). Given that *X. dendrorhous* exhibited different growth rates on different carbon sources, we normalized the data by dividing fluorescence by OD_600_ (optical density at 600 nm). Additionally, we used the *X. dendrorhous* native strain cultured in the same conditions as control to omit autofluorescence. This value is reported in normalized relative fluorescence units (RFU) and is calculated according to the following equation:

$$Normalized\;RFU =\frac{{Fluorescence}_{mutant\;strain}}{{OD}_{mutant\;strain}}-\frac{{Fluorescence}_{native\;strain}}{{OD}_{native\;strain}}$$

In the initial screening, only the strain carrying the *LAD* promoter showed clear inducibility. Consequently, the remaining four engineered strains were excluded from further experimentation. That is, only the *LAD* promoter was considered in subsequent experiments for characterization purposes. The dose-response relationship was evaluated at 48 h in 24-well plates with different concentrations of arabinose (0.05, 0.1, 0.25, 0.5, 0.75, 1, 2.5, 5, 10, 20, and 30 g/L). Fluorescence development over time for the *LAD* promoter was determined in Erlenmeyer flasks (250 mL) containing 50 mL of induction/repression medium (sampling 1 mL was performed as needed). The two-stage culture was performed in Erlenmeyer flasks (250 mL) containing 7.5 g/L glucose and 7.5 g/L arabinose (Fig. 1c). Samples were taken as required, and sugar concentrations were determined by HPLC as described by Rangel-Basto et al. (2024). In all these experiments, samples were pretreated as described above, and the data were analyzed by dividing fluorescence by OD_600_.

## Results

### Identification and evaluation of inducible promoters

To identify likely inducible promoters in *X. dendrorhous*, we reviewed the literature on promoters previously reported in other yeast species and on genes that potentially have well-regulated promoters. We located these genes and conducted a BLASTp analysis to identify orthologs in *X dendrorhous*. Among the genes potentially controlled by an inducible promoter, we focused on carbon-source-induced promoters because we aimed to identify promoters suitable for use in complex media to facilitate their scale-up. Conversely, we decided to exclude for future work those that require a chemically defined medium for their induction, for example, promoters induced or repressed by the copper concentration (*CRT3*,* CTR31*,* CCC2*), methionine (*MET16*), thiamine (*THI4*,* THI5*), or ammonium (*NAR1*). Seven putative carbon-source-induced promoters were detected, that is, *GAL1*, *GAL7*, *GUT*, *ICL*, *LAD*, *AG*, and *XDH*. Despite being present in the *X. dendrorhous* genome, *GAL1* and *GAL7* were excluded from further studies due to the known yeast incapacity to metabolize their substrate. Each potential promoter region of the five remaining promoters was delimited and amplified by PCR using appropriate primers. Subsequently, the amplicons were cloned into the pXDrDNA-EYFP plasmid, which possesses a Type IIS cloning site upstream of the *EYFP* gene. After cloning each promoter separately into the plasmid, *X. dendrorhous* cells were transformed, and clones containing the sequence encoding *EYFP* were selected for further study.

**Fig. 2:**
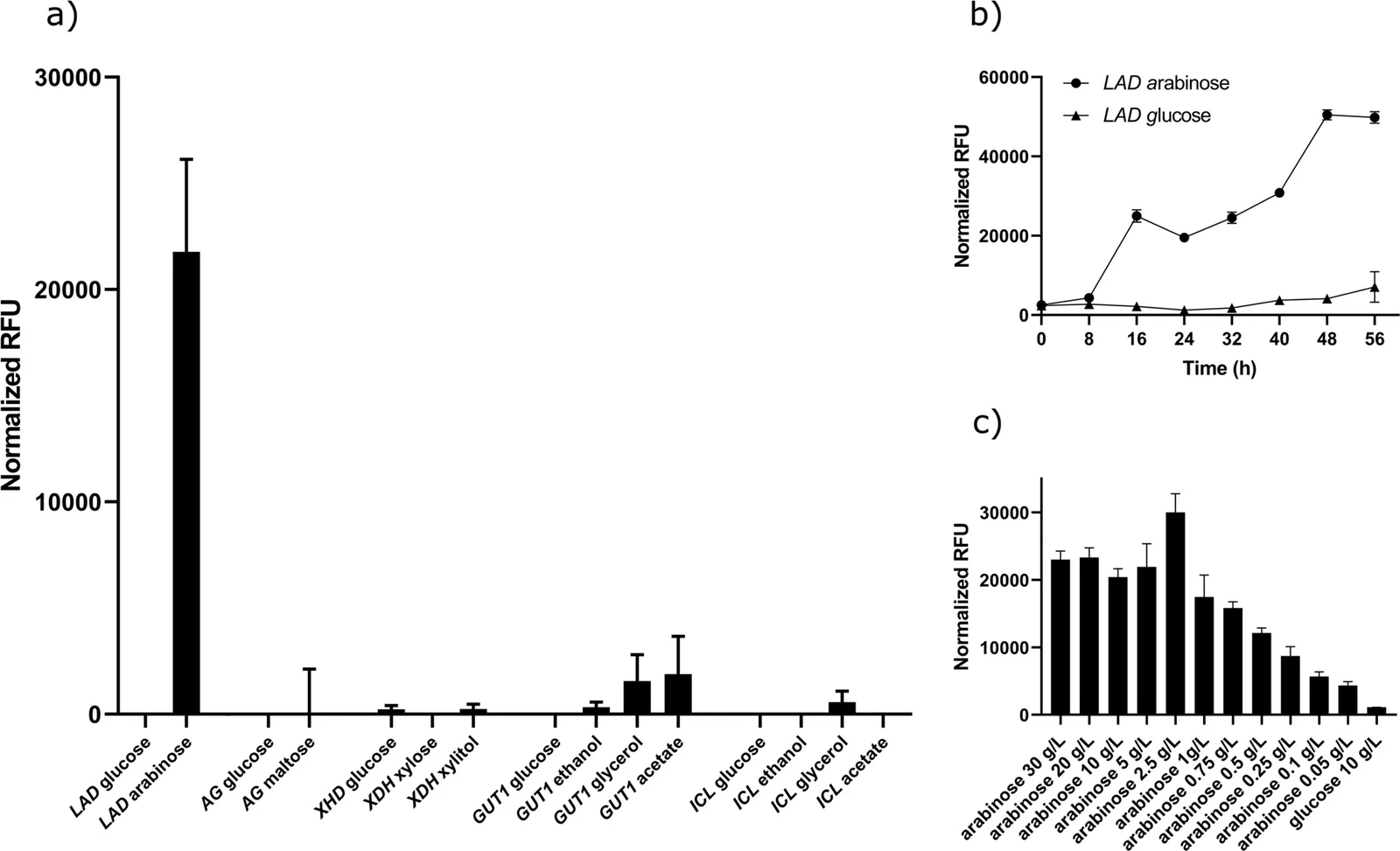
Evaluation and characterization of inducible promoters of *X. dendrorhous*. **a** Screening of candidate promoters in microplate culture. Panel **a** shows the fluorescence of *X. dendrorhous* cells harboring the studied promoters. Fluorescence was normalized to the cell density and corrected for autofluorescence of the native strain. **b** Time response of the LAD promoter. Fluorescence over time of *X. dendrorhous* cells harboring the LAD promoter under induction/repression (56 h). **c** Dose–response of the LAD promoter in the engineered *X. dendrorhous* strain. The evaluation was done with increasing concentrations of arabinose, and glucose at 10g/l as a repression control. Values are means of biological replicates with standard deviation shown (*n* = 3)

All *X. dendrorhous* strains were grown under repression/induction conditions using different substrates as detailed in Table 1. Among the evaluated strains, only the one carrying the *LAD* promoter showed marked fluorescence (Fig. 2a), so we next characterized its temporal and dose response.

**Table 1:** Putative inducible promoters studied in *X. dendrorhous*. After reviewing the literature, a total of five inducible promoters were selected for evaluation in *X. dendrorhous*. These promoters have previously been reported as inducible; each is induced by a specific substrate and repressed in glucose

Promoter	Repression conditions	Induction conditions	Organism	Reference
*GUTI* (glycerol kinase)	Glucose	Ethanol, glycerol, or acetate	*S. cerevisiae*	Grauslund et al. (1999)
*ICL* (isocitrate lyase)	Glucose	Ethanol, glycerol, or acetate	*S. cerevisiae*	Teranishi et al. (1994)
*LAD* (L-arabinitol 4-dehydrogenase)	Glucose	Arabinose	*Trichoderma reesei*	Richard et al. (2001)
*AG* (alpha glucosidase)	Glucose	Maltose	*Hansenula polymorpha*	Alamae et al. (2003)
*XDH* (xylitol dehydrogenase)	Glucose	Xylitol or xylose	*P. stipitis*	Bicho et al. (1988)

### Time and dosage response of the LAD promoter

*X. dendrorhous* cells carrying the *LAD* promoter were grown under both induction and repression conditions. Figure 2b shows a marked and time-dependent increase in fluorescence in cells carrying the *LAD* promoter up to 56 h. Furthermore, the relatively low fluorescence in the absence of arabinose suggests that *X. dendrorhous* cells with the *LAD* promoter exhibit strong transcriptional control.

On the other hand, we hypothesized that fluorescence could be modulated in response to the arabinose concentration in the medium. Consequently, *X. dendrorhous* cells carrying the *LAD* promoter were treated with increasing concentrations of arabinose to obtain information on the cellular response to arabinose dosage. The *LAD* promoter exhibited a dose-dependent response within the range of 0.05 and 1 g/L of arabinose, reaching saturation and maximal fluorescence at 2.5 g/L, where the fluorescence remained relatively stable up to 30 g/L (Fig. 2c). This dual behavior enables precise control over gene expression, confirming the *LAD* promoter as a versatile tool for biotechnological applications.

### *EYFP* expression in a two-stage culture

**Fig. 3:**
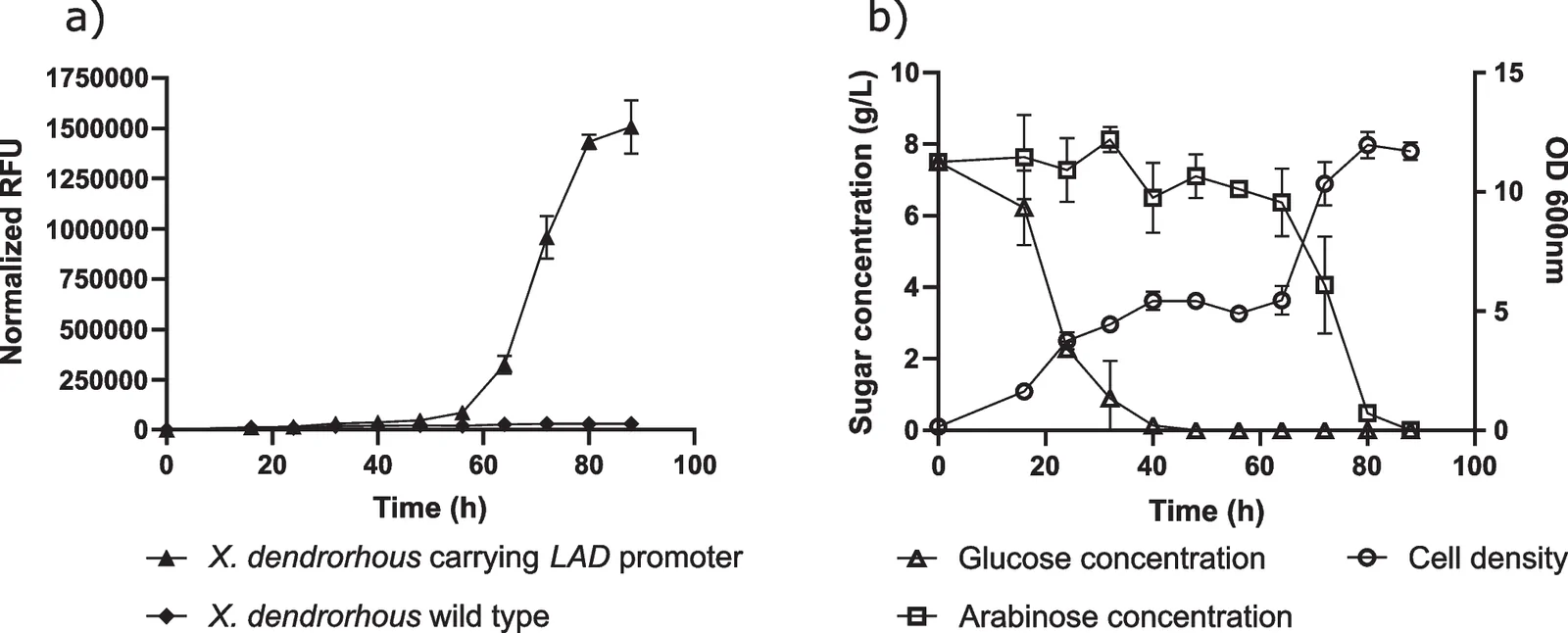
Two-stage culture of *X. dendrorhous*. **a** Comparison of fluorescence between *X. dendrorhous* harboring the LAD promoter and wild-type *X. dendrorhous*. Both were cultured in YM medium with the addition of (g/L): glucose 7.5 and arabinose 7.5. **b** Time course of sugar concentration and cell density of the engineered *X. dendrorhous* carrying LAD promoter. Values are biological replicates means with standard deviation shown (*n* = 3)

*X. dendrorhous* carrying the *LAD* promoter exhibited very low fluorescence under repression conditions and high fluorescence under induction conditions. That contrast made this strain an appealing candidate for exploring a two‑stage culture strategy. This approach has been successfully used for years to drive recombinant protein production in *P. pastoris*. To implement it, we grew the engineered *X. dendrorhous* strain in a medium containing two carbon sources, i.e., arabinose and glucose. By providing both sugars from the start, we could avoid the need to add the inducer at a precise time and instead let the cells themselves determine the transition. This design relied on the assumption that arabinose would be consumed only after glucose was fully depleted from the medium. Once that shift occurred, *EYFP* expression was expected to be primarily associated with arabinose metabolism. Firstly, the *X. dendrorhous* carrying *LAD* promoter and the native strains were grown both in YM medium added with glucose 7.5 and arabinose 7.5 (g/L). The engineered *X. dendrorhous* cells showed low fluorescence levels during the early 56 h, but afterwards, fluorescence increased markedly. In contrast, the native cells showed very low fluorescence throughout the entire culture period. These results indicate that *EYFP* expression, driven by the *LAD* promoter, effectively contributes to the fluorescence emitted by the engineered *X. dendrorhous* cells. In addition, Fig. 3a shows that the marked fluorescence increase beyond 56 h was clearly associated with the shift from glucose to arabinose use. As we expected glucose was consumed first, followed by arabinose; however, arabinose consumption did not begin immediately upon glucose depletion. Instead, an adaptive period from 40 to 64 h was observed, during which arabinose remained practically constant (Fig. 3b). Correspondingly, OD measurements showed a typical diauxic growth pattern, that is, two growth phases corresponding to glucose and arabinose consumption, respectively, with an adaptative phase between them.

## Discussion

In this work, five inducible *X. dendrorhous* promoters were identified and evaluated using *EYFP* as a reporter. A modular plasmid was constructed, and each putative promoter was cloned using a Type IIS Golden Gate Assembly. *X. dendrorhous* native cells were transformed and grown on various carbon sources, and the fluorescence emitted by the cells was measured. As shown in Fig. 2a, among the transformants obtained, only the one carrying the *LAD* promoter showed high fluorescence under induction conditions. Thus, this research focused on the characterization and practical utilization of this promoter. In experiments with the *X. dendrorhous* carrying the *LAD* promoter under glucose repression conditions, minimal fluorescence leakage was observed, suggesting strong transcriptional control. It is well known that at least some carotenogenic genes in *X. dendrorhous* are under the control of the Mig1 repressor (Alcaíno et al. 2016). Besides, the main genes required for the metabolism of galactose, maltose, and sucrose in yeast are also regulated by Mig1 (Weinhandl et al. 2014). Notably, the Mig1 repressor binds to DNA fragments containing Mig1 boxes*.* Thus, the consensus sequence SYGGRG and other Mig1 boxes previously reported were searched within the *LAD* promoter sequence. The promoter region length, 483 pb, contains two Mig1 boxes at positions −182 to −177 and −159 to −155. However, no TATA box was found within the promoter sequence. Based on the leakiness less fluorescence, and the above cited Mig1 boxes, our results suggest that the *LAD* promoter could be regulated by Mig1. However, further experiments are required to confirm this hypothesis and precisely delineate the regulatory mechanism.

Since one of the objectives of this research was to find inducible promoters for eventual practical applications, we analyzed the dose-response relationship and the time-dependence of the expression driven by this promoter. *X. dendrorhous* cells carrying the *LAD* promoter showed a relatively steady expression of *EYFP* during the entire culture period. Since this is the first carbon source-inducible promoter described in this yeast, we were unable to compare its time-dependence behavior with that of other *X. dendrorhous* promoters. However, compared to the strong promoters described by Hara et al (2014b, a), the behavior was similar, i.e., fluorescence steadily increased throughout the culture. Similarly, the *NAR1*, *ICL1*, and *CTR3* promoters of the basidiomycete yeast *R. toruloides* exhibited a similar profile (Johns et al. 2016). Furthermore, our dose-response analysis indicates that *LAD* promoter-driven *EYFP* expression can be finely modulated by arabinose concentration. One advantage of a dose-sensitive promoter is that it allows for exceptional modulation of gene expression, though a high concentration of the inducer is often required to achieve maximal expression. In contrast, a promoter like *LAD* allows for high levels of expression even with relatively small amounts of the inducer.

The practical utility of the *LAD* promoter was validated in a two-stage culture. This cultivation method, which has proven effective with *E. coli*, *S. cerevisiae*, and *P. pastoris*, is typically used when detrimental proteins to microbial cells are expressed. *X. dendrorhous* has not yet been used for recombinant protein expression. However, two-stage cultures may be suitable for ensuing metabolic engineering applications (Weinhandl et al. 2014), particularly, for metabolic pathway engineering where inducible promoters allowing the control of key biosynthetic steps are required to redirect carbon flux. In this work, *EYFP* was expressed in a two-stage culture as a marker of gene expression; however, an analogous strategy could be easily applied to other proteins. Given the highly active acetyl-CoA metabolism in *X. dendrorhous* and the variety of metabolites derived from this molecule, this scheme could well be useful in metabolic engineering to generate yeasts that are hyperproducers of terpenoids, polyunsaturated fatty acids, and other compounds (Miao et al. 2011; Misawa 2021).

A key finding in this work is that, to our knowledge, no reports have been published on native arabinose-induced promoters in yeast. Species such as *S. cerevisiae* or *Pichia pastoris* are, in fact, unable to metabolize arabinose naturally. Although yeasts like *Candida tropicalis*, *Kluyveromyces marxianus*, or *Scheffersomyces stipitis* possess this ability, arabinose-inducible promoters have not yet been described for any of them. Therefore, our work is the first to reveal an arabinose-inducible promoter in yeast, providing a novel genetic tool. Beyond this discovery, arabinose has some advantages as an inductor for genetic control. Arabinose has previously been reported to stimulate astaxanthin accumulation in *X. dendrorhous* without causing toxic or detrimental effects on growth, like ethanol or acetate. (Rangel-Basto et al. 2024; Vázquez et al. 1997; Yamane et al. 1997). Moreover, arabinose is a commonly occurring sugar in nature (Rangel-Basto et al. 2024), being so, saccharified sugarcane bagasse, or other low-cost hydrolysates from lignocellulosic materials, can be suitable carbon sources to grow *X. dendrorhous*, or for induction of recombinant protein expression. Considering the varied *X. dendrorhous* abilities to metabolize carbon sources, synthetize acetyl-CoA-derived compounds and its amenable genetic manipulation, the *LAD* promoter disclosed in this work represents a valuable tool to take advantage of its metabolism as a cell factory for a sustainable production in the biotechnological field.

## Supplementary Information

Below is the link to the electronic supplementary material.

ESM 1 (377 KB DOCX)

## Data Availability

All data in this study, including primers, promoter sequences, and plasmids, can be found within the paper and its Supplementary Information.
