# Supplementary material for: A novel arabinose-inducible promoter in the carotenogenic yeast Xanthophyllomyces dendrorhous
Source: Appl Microbiol Biotechnol. 2026 Jul 23;110:312. doi: 10.1007/s00253-026-13957-0 (PMC13680592; doi:10.1007/s00253-026-13957-0)
Supplement: Supplementary file 1 [file 253_2026_13957_MOESM1_ESM.docx]

**Applied Microbiology and Biotechnology**

**Supplementary material for:**

**Identification of a new arabinose-inducible promoter in the carotenogenic yeast *Xanthophyllomyces dendrorhous***

Author information

Oscar U. Garcia-$\mathrm{Flores}^{1}$, Rodolfo $\mathrm{Marsch}^{1},$Alejandra Sánchez-$\mathrm{Licona}^{1}$, Teresa Ponce-Noyola^1^, Luis B. Flores-$\mathrm{Cotera}^{1}$

Address information

1 Department of Biotechnology and Bioengineering, Cinvestav, Av. Instituto Politécnico Nacional 2508, Col. San Pedro Zacatenco, México City 07360, México.

Correspondence should be addressed to: Luis B. Flores Cotera. E-mail: [lfcotera@cinvestav.mx](mailto:lfcotera@cinvestav.mx)

**Table of contents**

**Supplemental Table S1: Primers used in this research…………………………………………………………………2**

**Supplemental Table S2: Delimited sequence of inducible promoters……………………………………………3**

**Supplemental Table S3: Description of plasmid pXDrDNA …………………………………………………………..5**

**Supplemental Figure S1: Verification of the transformation by PCR…………………………………….6**

**Supplemental Tables**

**Table S1. Primers used in this research.**

| **Primer name** | **Sequence 5´ 🡪 3´** |
| --- | --- |
| LAD F | GTACATGGTCTCCTAGAAGATGAGGGAGGATGTACAGAGAGG |
| LAD R | TACATGGGTCTCCACCATGGTGACGGATTTGTCTGAG |
| ICL F | GTACATGGTCTCCTAGAAGGAACGAGCTGAACGTAGTTCAC |
| ICL R | TACATGGGTCTCCACCATACATGATGAAAAACAGACGACTGTGA |
| AG F | GTACATCGTCTCCTAGAACATCAGGTGGCCTGAGCTGAA |
| AG R | TACATGCGTCTCCACCATATCTGGGTGATGTACGATGAAGAGC |
| GUT F | GTACATCGTCTCCTAGAACTTTCTCGGACGGTCAACGGA |
| GUT R | TACATGCGTCTCCACCATAGTATCTGGACAGGACTGGGGA |
| XDH F | GTACATGGTCTCCTAGAAGAAATGTTCCGATGATCATCATATATG |
| XDH R | TACATGGGTCTCCACCATGATAGCGATAAGAGTAAGTGCT |
| EYFP F | AGTAGAAGAGACCAAATCAAGGTCTCATGGTGAGCAAGGGCGAG |
| EYFP R | AATGTGAGAGAGGCGGGGTAATTTACTTGTACAGCTCGTCCATGCC |
| GDH Terminator F | CGGCATGGACGAGCTGTACAAGTAAATTACCCCGCCTCTCTCACAT |
| GDH Terminator R | AGAGCGCGGATAGGATATACTTGTTTC |
| rDNA F | AATCATCGAATCTTTGAACGCACCTT |
| rDNA R | GCAAGAATTGCAATGCTCTATCCC |

**Table S2. Delimited sequence of inducible promoters.**

| **Gene promoter** | **Sequence 5´🡪 3´** | **Accession number** |
| --- | --- | --- |
| L-arabinitol dehydrogenase (*LAD*) | GATGAGGGAGGATGTACAGAGAGGATGGATATCAAGGTGGAGATAGAAATGTATGACAAGTCTCGAGACATCCAAGGTTTAACCTTCCTCTCGCCTCGCAACACCAACATCAGGCATGTTTGCCATTCTTCCTCTCTGTCCTTCCCTTCGACTTCTGGTCTGACCATATACAGCGCTTGTAGATCTAGACACACAGCAGACAGTGGACCACATCGACCCAGCCCTCATGTTGAAGGAGGAGGAGGAAGATAGACAACACAGACCAAGCTCGGCGCGGACACTTGCCTGATTGAGAATTTATCCCCGGCCGGTGGAATCTTCGGTCCGGAGCCGCAGAGGAAAGTCGTTAAAAACTCACGACCCAGCTCGTTGCTTGTGGTTCTCTTTCGTCTCTCTCTATCTCTCTATCCGTTTGTTCGAAAGCTCAAGAAAAGCAATCACCTCTCTCACCACTCAGAAGAAATCTCAGACAAATCCGTCACC | CED84470.1 |
| Isocitrate lyase (*ICL*) | GGAACGAGCTGAACGTAGTTCACTTGTGTTCGATGATTACCTGATCTTCCCGCGATTGGTGTTGTACCCTCTAGGCTATATCTTATTCTCTCGACAGACTACTTTGACCGTCTGTCTGCTCGGTTTGCTGGTTGTAATATGTCTTTCTCTATCAGTAGGCGACTTTCGAGATGTATTGCTTCTCAAGTCATGATTCATGCTTTCTTTTTCACGCTGGTACAGAGCTAGGAACAAACACACCTGATGACAACACTGTCCAAATCCAGAGTCTGCCAGACTCCTGTCATGAATAACACCTGACGAACCATGAAACTTGGTTAGGGTTGTTAAATGTTTAATTTCACTTGGTTCGGCTTCTATCACAATGTTCTCTTATGAAACTATCAATTCTGTATTTTCTCAAGGTTTTCTGGCCAGGCGGCACCAAGTTTCATCGAGTGTGTTATGCAATTTCATCGGACGATGATTGGTGTTCAGGTATCGTTTAACCGAGCCATACATGTCTTAATAAAAAAAAGGAATAAAAAATCTTTGAGTAGGTAGGCTTTTCCAAAAGTTACTTCCGAATCCAGCATGAAACATTGGTCGATGGATTTCGGACTGGGTCCGAACGTGTTCGGCCGTCAGCATCGGAAAGAATCGTTTCGAGCAGTAGAAGAACAGAAGCCAATGCAGGCCGAAGCAAACGAAATGTGTATCCACCCCTTGCAGCTGGGCTGGCAGAGCTCATCTGCTCACATGTTTCGTCTCGTTCTTCCGGCCAGAACGATTGTTCTCTCACTCACTGGCTTCCTGAAATACCCGAAGGCGTTCGAGCGAGTCAGCGAGCTAACCGGCGAAACTTACAGCTAGACACCCGATCATTCTCATCTCAGGTTCAATTCCAGGTTGACCGAGTGGTGTCAATCTGAACGAGTGGTGTCAACCCAGTCTCCTGTCCTTTTGACTCGTTAGGAACCAGTGGTGTACATCTTGGAGAGGCCATATATATGCTCCCTTTGCACGTATCCTCAATCGATTTTGTTTCTCTCTTCATCTCACAGAAAACCTATTTGAACCAACTGATTTCATCCAGAGAGTCACAGTCGTCTGTTTTTCATC | CED85129**.**1 |
| Alpha glucosidase (*AG*) | CATCAGGTGGCCTGAGCTGAACAAGCACGGGATGGATGACGACATGGGCGGTGTCGCCCTGCCTGCCCGACCGACGGGTGGTGCCGGTCTCCGAACGAATGCGCTGGACGGCGAAGATTCTGACGACGGAATGGATGGTCTGACGGACGAACGGGGAGAGCATCCCTCGAACAGATCCGAAATCTCGGATGTGTATGGCTACCCTGCCCAGATTCCGCCAGACGGAACGACCGGGGCCCACACTGGGTACTATGACGTTGATCCCTACGAACAGATCGATTCAAGAGGCTACCCCACTTCCAATACAGATATTCCTCTCTACAGGACCGATCAATCATCCGGCGCGCCAGACATGTACAGCACCGGGACCCACCAGCATGAGATCTATGGGTCGGCTGGTGGTCAGCAGGAGTCCTACCATGCAGGGGACTCGAGAGTCGGAGATGGAGGTGAGGAAGAGATTCCGATGAGTCAAGTCTCTAGTTCCGGGCCAAGAAATCCGTATCCGTCGTTGATGGGCGTACACGATCCGTATGGCTTGGGTGGTAGATCGGACAATCCGTACGGGGCGGCGGGAGTGTCCAGCAGTCGGATGGATGATAGGTACTGATCGTAGGGTGTGTGTGCTCTATGAGTTGAAGCTGGCCTTCATGAGGTTTCCTCTCTTATTGTTTTCCTTCTTTTCAACGGGACCCTCGTTTTCTTCCTGCTTTCTATACATCTTTCATAGTTTGTTAGCTCTGCTTCTATCCTCCTGTGCTCAAGGTGTCTGTTCTTTTTCTTATTCGTTCGGTTGATCGTATTGTATACCCCTTTCCTTCTAGTTGATTCGGTTTTCATATGGGGTATCTCTTCTTTTTTTTGGCCTCCTTCATCCGCTGTCTGAGAATGAAACTCATTCTGTTCACATAGCTCTTCTCTCCAGACCTTCTCTGTACGTTTGTGTGCATGCCGTCGACGCTCAAGAAGAGCACGCGATGGCCGATACATGTCTGAAACACATGAACCTGAATAGTTGGATGACGAACAGAACATGGACAAGGGGAAGCAAGACAATGTTGAATGAACCCAACCTTGTCGGCTGACCGGCAGATGAAGTTCGGTATGTTTTCTTGTTTGTCTTCTCGGTTGATCCCGCAGGGAATCGCGTCGAGCATGTTTACCAAGGAAACGTTGATCTTCTCGCCCTTGTTGGTTGAAGGAGTACTGGATTCGTCTTCGGTGGTTGACTCCGTCTGTCGGTTGTTGTTGCTGTTGCTGTTATTGAGGCTTGGCTATCGCTACTGCTGCTAGTGCTGATTATGCCAATGCTGTTGCTGCTCTTGGCTGTGGGCTGACCGACCCGTTGATATAAAAAGCGGTCGAATTTTGGAGTTTAAGTTCTTTCTTGTTCCTTCACAACCGTCTTCAGACCTGCTTCCTCTCTCTCTCGCTCTTCATCGTACATCACCCAGAT | AOR81603.1 |
| Glycerol kinase (*GUT1*) | CTTTCTCGGACGGTCAACGGATGGGATGAACCGAGCGAGTAGAGAAGAGAGAGCAGAGGAAGGTTGAGAGCACCGAGGGTTGCTTAGTAAGCAGAGAATTGGCCGAGAGTGTGACTGACACGTGAGTCTGAGCGGTGAAAGGCTACGCCGGCGAAGCCACACACGCTGTGCATGTCATAACCTCGTAGCCACTTGACGAGCGTTTCATAACCATCCATAACCATTCATGGTTAGCCTTGACCACATCATAACCACATCATAACCATTGTATAACCATTCTATAACCACCCCGTAACCACCGAGCCATACCCGGCTACAGCCCTGCCCGTAACCACGGGCCCCGCTCCGGCACGAAAGCGGACCGGGTTCACGCCCCGCCGACGTTCTGGGTGCTGCTCAATCTTGGTCTCCTTGCCATCTCCAACGACCATCAACTCATCAACCATCAGCCATAAACACATTCCCCAGTCCTGTCCAGATACT | KX384889.1 |
| Xylitol dehydrogenase (*XDH*) | GAAATGTTCCGATGATCATCATATATGTTGCTTGTAGAATCTTTCTTTCCGTTGATTATGATGATGTGATTTCAGAATACGATACATGCTTTGCTAAGGTGGCAGACGAAAGAGTATTTCCTGTTTGGGCAAACATCTCCGGTCGGCCCGGCCGCGCAGGTTTTCTCCGCGGTAGTCACACTCAACTCTCATCTTCTACTTAAGCCGCGCTTGTTCTGGATCTCCTTGATTCCATCTTGGGGTATTTCTTTCCCTTCTTCTTTCGTTCTTTCTTTCTTTCTACTCTTGAACATCACCCTCTCCGACATCATCCATATCTTCAGCACTTACTCTTATCGCTATC | CED85526.1 |

**Table S3. Description of plasmid pXDrDNA. The integrative plasmid pXDrDNA (total size: 5316 bp) was constructed and annotated as follows: rDNA1 (4555 -238), ori (347-935,** **←), Neo/KanR/promoter (1219-2151,** **←), rDNA2 (2343-2850), GDH Promoter (2851-3487, →), BleoR (3488 -3862, →), and Actin Terminator (3863-4536,** **→)**

**Sequence 5’🡪 3’**

ACTAGTTTAACCTGCTAAATAGCCCGGCCGGCTCTTGCTGGTCGCCGGCTTCTTAGAGGGACTATCAGCATCTAGCTGATGGAAGTTTGAGGCAATAACAGGTCTGTGATGCCCTTAGATGTTCTGGGCCGCACGCGCGCTACACTGACAGAGCCAGCGAGTTTCTTTCCTTGGCCGAAAGGTCTGGGTAATCTTGTGAAACTCTGTCGTGCTGGGGATAGAGCATTGCAATTCTTGCggcggtaaTACGGTTATCCACAGAATCAGGGGATAACGCAGGAAAGAACATGTGAGCAAAAGGCCAGCAAAAGGCCAGGAACCGTAAAAAGGCCGCGTTGCTGGCGTTTTTCCATAGGCTCCGCCCCCCTGACGAGCATCACAAAAATCGACGCTCAAGTCAGAGGTGGCGAAACCCGACAGGACTATAAAGATACCAGGCGTTTCCCCCTGGAAGCTCCCTCGTGCGCTCTCCTGTTCCGACCCTGCCGCTTACCGGATACCTGTCCGCCTTTCTCCCTTCGGGAAGCGTGGCGCTTTCTCAATGCTCACGCTGTAGGTATCTCAGTTCGGTGTAGGTCGTTCGCTCCAAGCTGGGCTGTGTGCACGAACCCCCCGTTCAGCCCGACCGCTGCGCCTTATCCGGTAACTATCGTCTTGAGTCCAACCCGGTAAGACACGACTTATCGCCACTGGCAGCAGCCACTGGTAACAGGATTAGCAGAGCGAGGTATGTAGGCGGTGCTACAGAGTTCTTGAAGTGGTGGCCTAACTACGGCTACACTAGAAGGACAGTATTTGGTATCTGCGCTCTGCTGAAGCCAGTTACCTTCGGAAAAAGAGTTGGTAGCTCTTGATCCGGCAAACAAACCACCGCTGGTAGCGGTGGTTTTTTTGTTTGCAAGCAGCAGATTACGCGCAGAAAAAAAGGATCTCAAGAAGATCCTTTGATCTTTTCTACGGGGTCTGACGCTCAGTGGAACGAAAACTCACGTTAAGGGATTTTGGTCATGAGATTATCAAAAAGGATCTTCACCTAGATCCTTTTGGGGTGGGCGAAGAACTCCAGCATGAGATCCCCGCGCTGGAGGATCATCCAGCCGGCGTCCCGGAAAACGATTCCGAAGCCCAACCTTTCATAGAAGGCGGCGGTGGAATCGAAATCTCGTGATGGCAGGTTGGGCGTCGCTTGGTCGGTCATTTCGAACCCCAGAGTCCCGCTCAGAAGAACTCGTCAAGAAGGCGATAGAAGGCGATGCGCTGCGAATCGGGAGCGGCGATACCGTAAAGCACGAGGAAGCGGTCAGCCCATTCGCCGCCAAGCTCTTCAGCAATATCACGGGTAGCCAACGCTATGTCCTGATAGCGGTCCGCCACACCCAGCCGGCCACAGTCGATGAATCCAGAAAAGCGGCCATTTTCCACCATGATATTCGGCAAGCAGGCATCGCCATGGGTCACGACGAGATCCTCGCCGTCGGGCATCCGCGCCTTGAGCCTGGCGAACAGTTCGGCTGGCGCGAGCCCCTGATGCTCTTCGTCCAGATCATCCTGATCGACAAGACCGGCTTCCATCCGAGTACGTGCTCGCTCGATGCGATGTTTCGCTTGGTGGTCGAATGGGCAGGTAGCCGGATCAAGCGTATGCAGCCGCCGCATTGCATCAGCCATGATGGATACTTTCTCGGCAGGAGCAAGGTGAGATGACAGGAGATCCTGCCCCGGCACTTCGCCCAATAGCAGCCAGTCCCTTCCCGCTTCAGTGACAACGTCGAGCACAGCTGCGCAAGGAACGCCCGTCGTGGCCAGCCACGATAGCCGCGCTGCCTCGTCTTGGAGTTCATTCAGGGCACCGGACAGGTCGGTCTTGACAAAAAGAACCGGGCGCCCCTGCGCTGACAGCCGGAACACGGCGGCATCAGAGCAGCCGATTGTCTGTTGTGCCCAGTCATAGCCGAATAGCCTCTCCACCCAAGCGGCCGGAGAACCTGCGTGCAATCCATCTTGTTCAATCATGCGAAACGATCCTCATCCTGTCTCTTGATCAGATCTTGATCCCCTGGGCCATCAGATCCTTGGCGGCAAGAAAGCCATCCAGTTTACTTTGCAGGGCTTCCCAACCTTACCAGAGGGCGCCCCAGCTGGCAATTCCGGTTCGCTTGCTGTCCATAAAACCGCCCAGTCTAGCTATCGCCATGTAAGCCCACTGCAAGCTACCTGCTTTCTCTTTGCGCTTGCGTTTTCCCTTGTCCAGATAGCCCAGTAGCTGACATTCATCCGGGGTCAGCACCGTTTCTGCGGACTGGCTTTCTACGTGTTCCGCTTCCTTTAGCAGCtcttcaacgAGGAATTCAGTGAATCATCGAATCTTTGAACGCACCTTGCGCTCCTTGGTATTCCGAGGAGCATGCCTGTTTGAGTGTCATGAAACACTCTCAACCGGGTGTCCTTTCTTTGTTGAGAGGCGCCTGGCTTGGACTTGGACGGTTCTGCTGGCCCTGATTTGGTTCGGGGGTCGGCTCGTCTTAAAACGATCAGTGGGTGTGTGCGTCTGTGCTGGTGAAGCGCGGGCGGTGCCTGGACATGATAAGAAATTGTCGTCGAGGTGCTGTCTGTGTGTGTGTGTGCTTTTTTCGGAAGGCATGTGCTAGCCGGTGCGACTGTGACGCATGCGAACTGCTTCGAACCGATACTGTGTGGCCTCCTTTCGGGGGGGCGATGCACGTTTTTCTGATCGGGCCTCAAATCAGGTAGGACTACCCGCTGAACTTAAGCATATCAATAAGCGGAGGAAAAGAAACTAACCAGGATTCCCCTAGTAACGGCGAGTGAAGCGGGATGAGCTCAAATTTGTATGGCCAACACAGCAAGCGAGCTAGCCTACTGATCTCTTCCTTGGCCAAGATCCAGAAGACTGGATAGGTTAATTGATTGAACGGGAGAGTGCAAGACATTAACCCTAACAAGGGGTGAGTACCTGAACTAACCAGCGTGCTGCCAAGACACGTGATATATGGCTGTACGAAAAAGTCCGAGAACCTGAAAGAACATCCGAGAACGTCGGAGGATGCCGATGCGGATGCCGCGCACGGACATTGGGAAACACGAGCGGAGCGACCGAGGGGCGACCAGGGGGGGGGACGGAGGAGTGTGTGAGGAGGAGTAGGACTGAGGAGTGTGTGTGCGAACGGGGCCTGGTGTGTAAGCCCCAGGGACGGCTCAGGCGCTTCCCAGGCACTTTCAGGGGCACCCAGGCACGGAGATATGAAATCAACGATCGACCCAACTGATAAGGAAATTTGCAAATCGCCCTGATCTCGTTCCTGACTGCGCAGTTTTTCTTTCCCGTCCCTTTTTTCCCATATTTTATGTTTGCCCTGCCATCTTTTTTCCTCTTCCTCCCTTTTTCTCTTTCTCCACCATCTTCATCCTTCCCATCCCATCTCATCTATCTCCCATCTCACCTTCTCCTCCACATCTTCCATCAACCATGGCCAAGTTGACCAGTGCCGTTCCGGTGCTCACCGCGCGCGACGTCGCCGGAGCGGTCGAGTTCTGGACCGACCGGCTCGGGTTCTCCCGGGACTTCGTGGAGGACGACTTCGCCGGTGTGGTCCGGGACGACGTGACCCTGTTCATCAGCGCGGTCCAGGACCAGGTGGTGCCGGACAACACCCTGGCCTGGGTGTGGGTGCGCGGCCTGGACGAGCTGTACGCCGAGTGGTCGGAGGTCGTGTCCACGAACTTCCGGGACGCCTCCGGGCCGGCCATGACCGAGATCGGCGAGCAGCCGTGGGGGCGGGAGTTCGCCCTGCGCGACCCGGCCGGCAACTGCGTGCACTTCGTGGCCGAGGAGCAGGACTGAgtcaacaaagtctttctatcgggcggtttcgaacggtttcttttcttacgatagaggcacccttggcggtctttcgccagcggtggttttctcactctttttccaatattttcaccgacgtttttgctcgcgtgtatgtttttctctacttgatgctatcaatatcatggcttttacgttccgttgtattatcatcttctgcgcccttctgactgagatatcgaaatcgttacaacacaagacatataaaaatgagatttattcaactactaaataacgatacattagacgtaataaatagattcatagacaggcccgaatgttgatcgatcaaagactttaaacgtaattgcgacattctccaaaaccccaagccgaactctttccatcacctatttgttcccaaatttcgaaacatccactctctttctgaagctcagtatcacctcgagaacggatcttcaatctttagggcgtgtctttttccggtcttatcttgaatttgttatcgtcattcgatatagatctcccttattgtattctatccttcgaacgcgttttatacgtatcaaccctcgaccatgccgtccgattcatcccatcgagcctcacgatctggttcatcagacaagaacaggtcgaatacaaacggtccatcagctcagtcttcgtCTAGAGTTTAAACGCGGCCGCAGCAGCCGCGGTAATTCCAGCTCCAATAGCGTATATTAAAGTTGTTGCAGTTAAAAAGCTCGTAGTTGAACTTCAGGCCTGGTCGGCCGGTCCGCCTCACGGTGTGTACTGGTCGGCCGGGCCTTACCTCTTGGTGAGCCGTTCGGTCGTTTACGCGGCCGGGCGGGGAACCAGGATTTTTACTTTGAAAAAATTAGAGTGTTCAAAGCAGGCCTATGCCCGAATACATTAGCATGGAATAATAGAATAGGACGTGCGGTTCTATTTTGTTGGTTTCTAGGATCGCCGTAATGATTAATAGGGATAGTTGGGGGCATTAGTATTCAGTTGCTAGAGGTGAAATTCTTGGATTTACTGAAGACTAACTACTGCGAAAGCATTTGCCAAGGATGTTTTCATTAATCAAGAACGAAGGTTAGGGGATCGAAAACGATCAGATACCGTTGTAGTCTTAACAGTAAACTATGCCGACTAGGGATCGGGCGATGTTCTCTTTTGACTCGCTCGGCACCTTACGAGAAATCAAAGTCTTTGGGTTCTGGGGGGAGTATGGTCGCAAGGCTGAAACTTAAAGGAATTGACGGAAGGGCACCACCAGGAGTGGAGCCTGCGGCTTAATTTGACTCAACACGGGGAAACTCACCAGGTCCAGACAATATAAGGATTGACAGATTGATAGCTCTTTCTTGATTTATTGGGTGGTGGTGCATGGCCGTTCTTAGTTGGTGGAGTGATTTGTCTGGTTAATTCCGATAACGAAC

**Supplemental figures**

1. 1 kb Plus DNA Ladder
2. *X. dendrorhous* carrying *AG* promoter
3. *X. dendrorhous* carrying *GUT* promoter
4. *X. dendrorhous* carrying *ICL* promoter
5. *X. dendrorhous* carrying *LAD* promoter
6. *X. dendrorhous* carrying *XDH* promoter

**1	2	3	4	5	6**

1 000 pb

700 pb

**Figure. S1 Electrophoretic analysis of engineered strains. EYFP was amplified using the primer pair EYFP F/R to obtain a 768pb product.**
